# Communication openness and nosocomial infection reporting: the mediating role of team cohesion

**DOI:** 10.1186/s12913-022-08646-3

**Published:** 2022-11-24

**Authors:** Tiantian Yu, Xinping Zhang, Qianning Wang, Feiyang Zheng, Lu Wang

**Affiliations:** School of Medicine and Health Management, Tongji Medical School, Huazhong University of Science and Technology, Wuhan, Hubei China

**Keywords:** Communication openness, Team cohesion, Nosocomial infection reporting, Mediating effect

## Abstract

**Background:**

The states of IPC (Infection Prevention and Control) is serious under the COVID-19 pandemic. Nosocomial infection reporting is of great significance to transparent management of IPC in regard to the COVID-19 pandemic. We aimed to explore the relationship between communication openness and nosocomial infection reporting, explore the mediating effect of team cohesion in the two, and provide evidence-based organizational perspective for improving IPC management in the hospitals.

**Method:**

A questionnaire was used to collect data on communication openness, team cohesion and nosocomial infection reporting in 3512 medical staff from 239 hospitals in Hubei, China. Structural Equation Model (SEM) was conducted to examine the hypothetical model.

**Result:**

Communication openness was positively related to nosocomial infection reporting (β = 0.540, p < 0.001), and was positively related to team cohesion (β = 0.887, p < 0.001). Team cohesion was positively related to nosocomial infection reporting (β = 0.328, p < 0.001). The partial mediating effect of team cohesion was significant (β = 0.291, SE = 0.055, 95% CI = [ 0.178,0.392 ]), making up 35.02% of total effect.

**Conclusion:**

Communication openness was not only positively related to nosocomial infection reporting. Team cohesion can be regarded as a mediator between communication openness and nosocomial infection reporting. It implies that strengthening communication openness and team cohesion is the strategy to promote IPC management from the new organizational perspective.

## Background

Safety incidents reporting is extensively practiced internationally in health field. Recently a typical reporting was 31,434 cases of adverse events on COVID-19 vaccination reported within five month [1]. Prominent routine reporting includes the vaccine adverse event reporting system [2], the ADR (adverse drug reaction) reporting system [3], and the nosocomial infection surveillance system [4]. These reporting systems are in alignment with *the World Health Organization (WHO)*’ advocacy on the establishment of an international medical adverse event reporting system and emphasizing the importance of integrating adverse event reporting practice into hospital safety culture, which is of great significance to transparent management and so on [5]. For instance, the number of COVID-19 infections and deaths was reported daily from the *Johns Hopkins University* and weekly from *WHO*, greatly promoting IPC (infection prevention control) behavior in multiple countries and regions. Especially at the early stage of COVID-19, the number of infected healthcare workers was up to 1706 cases, accounting for approximately 20% of all infection cases [6]. Related study and policy advocacy showed that nosocomial infections surveillance and reporting could effectively reduce infection rates [7]. Moreover, efficient and reliable surveillance and reporting are vital for monitoring public health trends and early detection of disease outbreaks [8]. So the significance of nosocomial infection reporting is particularly prominent, especially how to promoting the reporting [9]. In this study, we focused on the procedure, timeliness and feedback of medical staff on reporting nosocomial events as indicators to promote nosocomial infection reporting.

There is some evidence of impact on reporting. For instance, qualitative interviews found that the lack of professional knowledge of nurses and imperfect reporting procedure had a negative impact on reporting in the ADR field [10]. And good management ability has a positive impact on reporting in the field of economic management [11]. In IPC field, quantitative analysis showed that electronic reporting system had positive effect on reporting [12]. But the organizational perspective is less concerned in the influencing factors of IPC reporting, especially communication openness and team cohesion. These two factors have important research value in improving performance and promoting innovation in the fields of public management and organizational management [13, 14]. Therefore, exploring the mechanism of communication openness and team cohesion in the nosocomial infection reporting may provide valuable insight for the improvement of nosocomial infection reporting and the IPC management.

Communication openness refers to the free willingness of medical staff to exchange, share, feedback and communicate information on IPC issues [15–17]. Unfortunately, less studies on the direct relationship between communication openness and nosocomial infection reporting has been published [18–20]. A survey attributed the low frequency of adverse events reporting to communication openness, possibly due to lack of understanding of the definition of adverse or infectious events and fear of punishment after reporting [18]. Similarly, quantitative study confirmed that good communication openness in hospital environment increased the frequency of medical error reporting [19]. Communication openness between medical staff in the intensive care unit helped to understand patient care goals, thus reducing the incidence of accidents and improving patient outcomes [20]. Based on the above review, it’s proposed that communication openness is positively related to nosocomial infection reporting (Hypothesis 1).

**Fig. 1:**
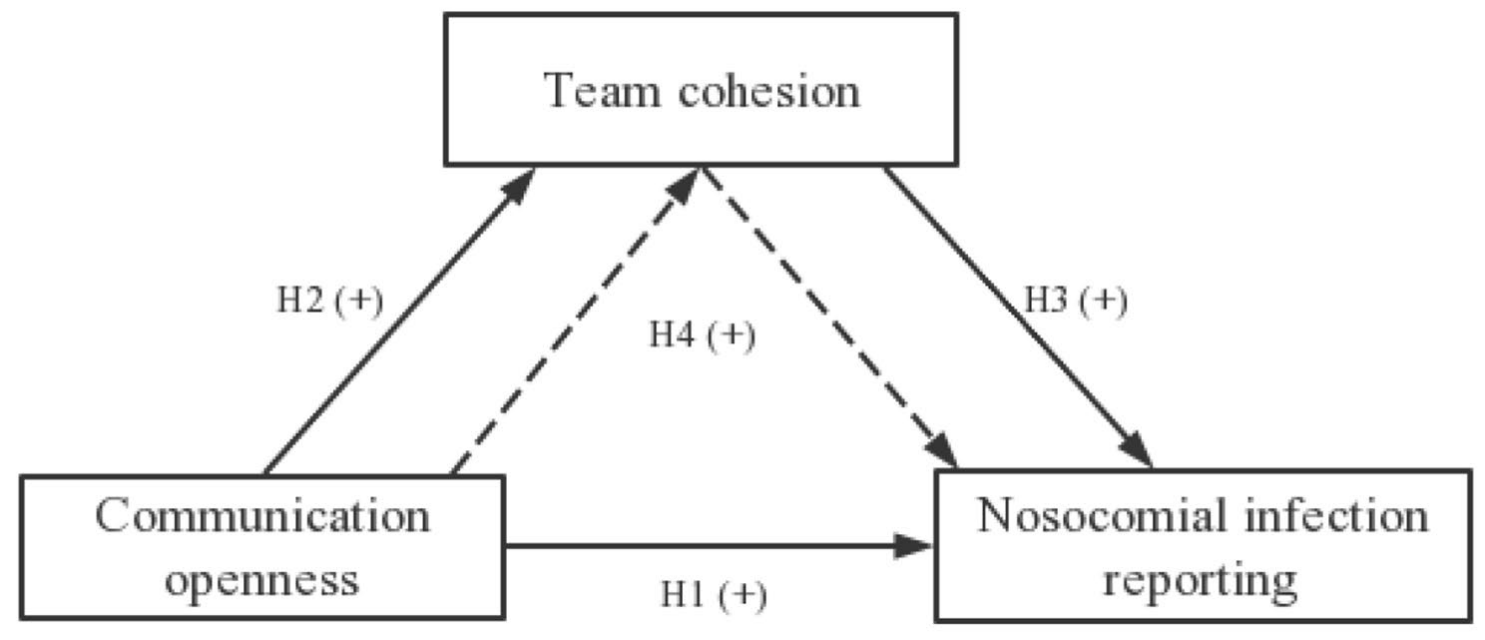
Hypothesized model of communication openness, team cohesion and nosocomial infection reporting

Team cohesion refers to an atmosphere of teamwork, mutual help and trust among medical staff [21, 22]. Van Woerkom and Sanders thought trust and cohesion increased when team members communicated and gave advice, and used quantitative evidence to reveal a positive relationship between cohesiveness and openness for sharing opinions and suggestions [23]. Highly cohesive teams experience less friction, greater trust and better interpersonal coordination [24]. Hence, the second hypothesis is that communication openness is positively related to team cohesion (Hypothesis 2). The view that effective team cooperation in health care can create a positive organizational atmosphere and improve treatment outcomes was supported [25]. Moreover, if teams were indeed working well together, they were subject to less internal pressure to speak up about infections thus improving adverse outcomes [26]. Based on the effect of team cohesion on healthy behaviors, we propose the third hypothesis that team cohesion is positively related to nosocomial infection reporting (Hypothesis 3). The aforementioned hypotheses form the theoretical model of the relationships among communication openness, team cohesion, and nosocomial infection reporting shown in Fig. 1. A study also proved that team cohesion moderated the relationship between team diversity climate and creativity, and team diversity climate indicated good team communication [27]. Moreover, cohesion had a significant partial mediating effect on health promotion behaviors [28]. Hence, in addition to direct effects, we propose that team cohesion serves as a mediator through which communication openness affects nosocomial infection reporting as well (Hypothesis 4).

## Methods

### Study design

A cross-sectional study was conducted through an online survey. The study data were anonymous to protect privacy. The pre-survey was adapted from Hospital Survey on Patient Safety Culture (HSOPSC) developed by Agency for Healthcare Research and Quality [29] and Leading a Culture of Quality in infection prevention (LCQ-IP) developed by Pogorzelsk-Maziarz [30], which were widely used in the field of patients safety culture and hospital infection. For example, " Staff will freely speak up if they see something that may negatively affect patient care” item in communication openness dimension from HSOPSC was corrected as “discuss publicly about nosocomial infection events” to make it suitable for nosocomial infection in this study. The questionnaire was formed which mainly included 3 dimensions (communication openness, team cohesion and nosocomial infection reporting) with 12 items, each of which were rated on a five-point scale (1 = completely disagree to 5 = completely agree).

### Participants

Participants were clinical medical staff. The response rate of participants was 100%. 3512 valid questionnaires of 239 hospitals were obtained with an effective rate of 92.25%. Informed consent was obtained from all survey participants. The inclusion criteria was that participants were on duty and were voluntary to participant in the survey anonymously.

### Quality control

Quality Control Center on Hospital Infection Management in Hubei province required clinical medical staff to fill the questionnaire. And the center sent the questionnaire to directors in hospitals. The directors were responsible for quality control by checking the questionnaire filling. The quality control before data analysis was based in the following inclusion criteria.

(1) Clinical medical staff’ working years were not less than 1 year.

(2) The time required to answer the questionnaire was not less than 3 mins (The minimum answer time tested by our research group was 5 mins).

### Measurements

**Fig. 2:**
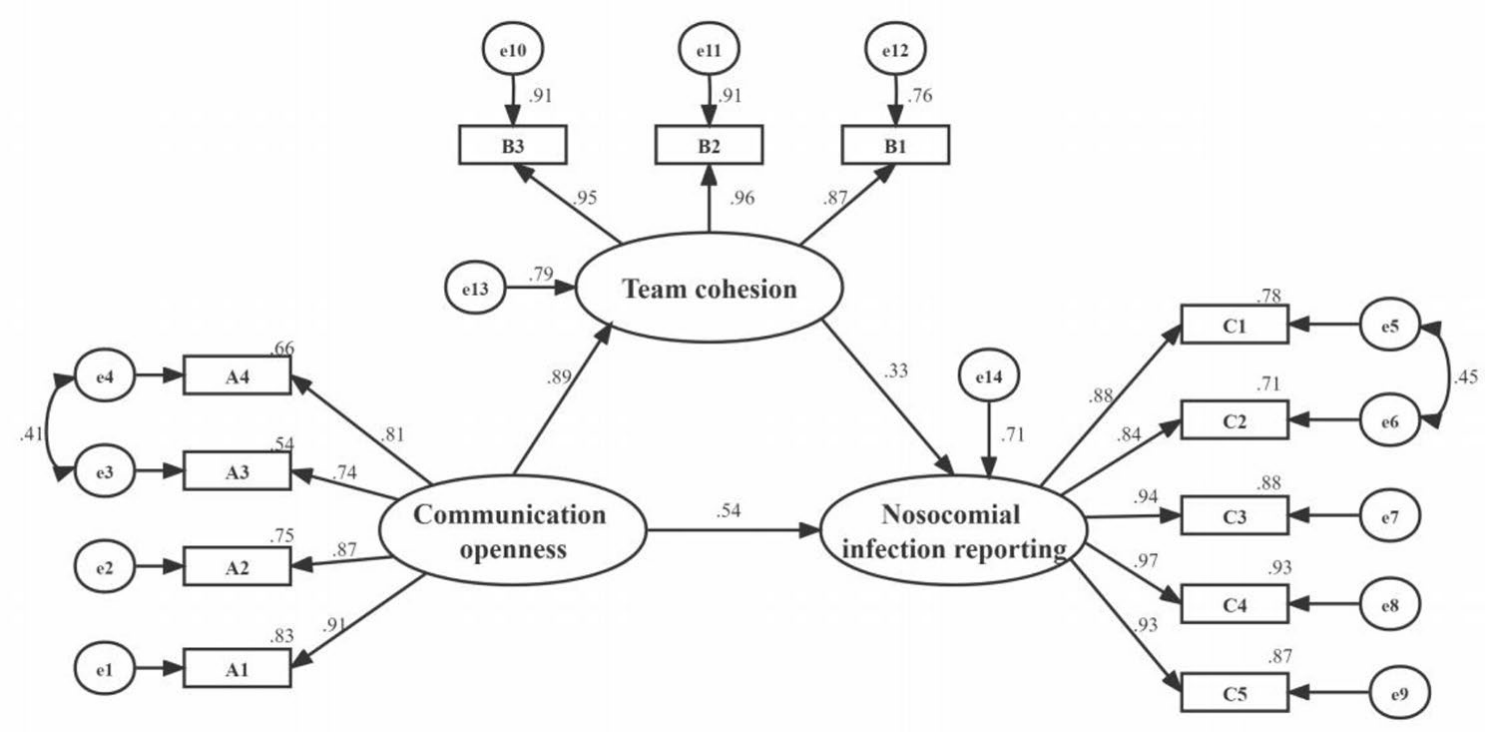
Results of the structural equation modeling of the effect of communication openness on nosocomial infection with team cohesion as a mediator. RMSEA = 0.068,RMR = 0.068, CFI = 0.984, NFI = 0.984, IFI = 0.984, RFI = 0.978;A1-A4 (the four items of communication openness), B1-B3 (the three items of team cohesion), C1-C5 (the five items of nosocomial infection reporting)

Communication openness was measured from 4 items including ‘talk freely about the hospital IPC measures’, ‘discuss publicly about nosocomial infection events’, ‘feedback the results of nosocomial infection events freely’, and ‘exchange and resolve the differences of opinions publicly’. In this study, Structural equation model (SEM) was conducted to assess construct validity, and the results confirmed that the factor loadings of the four indicator variables were no less than 0.74 (see Fig. 2), indicating acceptable construct validity. Cronbach’s α for communication openness was 0.909, indicating satisfactory internal consistency.

Team cohesion was measured from 3 items including ‘good coordination of carrying out IPC’, ‘work together to do IPC’, ‘departments to encourage team spirit’. The factor loadings of the three indicator variables were no less than 0.87 (see Fig. 2), indicating acceptable construct validity. Cronbach’s α for team cohesion was 0.946, indicating satisfactory internal consistency.

Nosocomial infection reporting was measured from 5 items including ‘establish a good reporting system’, ‘know how to report’, ‘timely report the vulnerabilities of nosocomial infection’, ‘immediately implement the measures after reporting’, ‘evaluate IPC results after reporting’. The factor loadings of the five indicator variables were no less than 0.84 (see Fig. 2), indicating acceptable construct validity. Cronbach’s α for nosocomial infection reporting was 0.958, indicating satisfactory internal consistency.

### Statistical analysis

The data were analyzed using SPSS (v. 19.0) and AMOS (v. 22.0) software. Descriptive statistics were performed to describe the demographic characteristics. T-tests and a one-way ANOVA were conducted to examine the differences in communication openness, team cohesion, and nosocomial infection reporting scores across demographic factors. A Pearson’s correlation coefficient was calculated to analyse the correlations between communication openness, nosocomial infection reporting and team cohesion. The effect of communication openness on nosocomial infection reporting via team cohesion was examined using a structural equation modelling with maximum likelihood estimation. The mediation effect test was carried out by using the bootstrap method. The goodness-of-fit of the model was evaluated with χ2 statistic, the Goodness of Fit Index (GFI), the Comparative Fit Index (CFI), the root mean square error of approximation (RMSEA), the Non-Normed Fit Index (NNFI) and the Incremental Fit Index (IFI). The model fitted well when GFI > 0.90, CFI > 0.90, RMSEA < 0.05, NNFI > 0.90 and IFI > 0.90.

## Results

Demographic characteristics of medical staff.

The mean age of medical staff was 34.69 (Standard Deviations = 8.28) years.The other demographic characteristic of medical staff are reported in Table 1.

**Table 1:** Demographic characteristic of medical staff and univariate analyses ( N = 3512)

Characteristic	Mean ± SD/ N (%)	Communication openness t/F	P	Team cohesion t/F	P	Nosocomial infection reporting t/F	P
**Age (year)**	34.69 ± 8.28	0.817	0.806	0.704	0.935	0.769	0.871
≤ 35	2213 (63.01)						
> 35	1299 (36.99)						
**Working time (years)**	8.25 ± 7.35	1.692	0.141	0.466	0.900	0.898	0.424
≤ 8	2214 (63.04)						
> 8	1298 (36.96)						
**Gender**		0.174	0.525	0.301	0.924	1.037	0.087
Male	561 (16.00)						
Female	2951 (84.00)						
**Occupation**							
Doctor	899 (25.60)	0.497	0.286	1.051	0.052	1.043	0.057
Nurse	2613 (74.40)						
**Professional Title**							
No	140 (3.99)	0.814	0.516	0.527	0.716	0.110	0.979
Primary	1791 (51.00)						
Medium-grade Sub-senior	1194 (34.00) 335 (9.53)						
Senior	52 (1.48)						
**Level of education**							
College	782 (22.27)	0.925	0.428	1.487	0.216	2.595	0.051
Undergraduate	2503 (72.26)						
Master	221 (6.10)						
Doctor	6 (0.17)						
**Clinical instructor**							
Yes	1474 (41.97)	0.501	0.479	2.508	0.113	2.252	0.134
No	2038 (50.03)						
**Department**							
Emergency	237 (6.79)	1.308	0.227	1.140	0.33	0.911	0.515
Surgery	801 (22.81)						
Internal Medicine	1312 (37.36)						
Chinese Traditional Medicine	47 (1.34)						
Anesthesia	43 (1.18)						
Rehabilitation	93 (2.64)						
Infection Disease	49 (1.40)						
Intensive Care Unit	168 (4.78)						
Gynecology and Obstetrics	258 (7.35)						
Ophthalmology and Otorhinolaryng-	103 (2.93)						
ology							
Pediatrics	401 (11.42)						

Univariate analyses of communication openness, team cohesion, and nosocomial infection reporting.

As shown in Table 1, univariate analyses indicated no significant differences in the communication openness, team cohesion, and nosocomial infection reporting scores for demographic characteristic.

Level of communication openness, team cohesion and nosocomial infection reporting and their correlations.

The level of above variables ranged from 4.5383.to 4.7139. Communication openness had both a significant positive relation with team cohesion (r = 0.807, p < 0.01) and nosocomial infection reporting (r = 0.793, p < 0.01), team cohesion showed a significant positive correlation with nosocomial infection reporting (r = 0.783, p < 0.01), as shown in Table 2.

**Table 2:** Mean, SD and correlations among communication openness, team cohesion and nosocomial infection reporting

	Mean	SD	Correlation coefficient		
			**1**	**2**	**3**
1.Communication openness	4.5383	0.66678	1		
2.Team cohesion	4.7139	0.58303	0.807^**^	1	
3.Nosocomial infection reporting	4.6644	0.56398	0.793^**^	0.783^**^	1

NOTE: **p < 0.01

### Analysis of the hypothetical model

The standardized estimates of the path coefficients for each variable are shown in Fig. 2. SEM revealed significant regression or correlation paths, and all beta path coefficients were statistically significant (p < 0.001). The SEM results indicated a good fit between our hypothesized model and the data (RMSEA = 0.068,RMR = 0.068, CFI = 0.984, NFI = 0.984, IFI = 0.984, RFI = 0.978).

### Mediation effect analysis of the hypothetical model

The results for the direct and indirect effects of communication openness on nosocomial infection reporting with team cohesion as mediators were presented in Table 3. Communication openness was positively related to nosocomial infection reporting (coefficient = 0.540, p < 0.001), and was positively related to team cohesion (coefficient = 0.887, p < 0.001). Team cohesion was positively related to infection report (coefficient = 0.328, p < 0.001). The mediating effect of team cohesion was significant (coefficient = 0.291, SE = 0.055, 95% CI = [ 0.178, 0.392 ]), making up 35.02% of total effect.

**Table 3:** Effects of communication openness on nosocomial reporting with team cohesion as a mediator

	Standard coefficient	S.E.	95% bias-corrected CI	Relative effect
Total effect	0.831	0.019	(0.791,0.864)***	100%
Direct effect	0.540	0.024	(0.450,0.643)***	64.98%
Mediating effect	0.291	0.055	(0.178,0.392)***	35.02%

Note: ***P < 0.001

## Discussion

The current study investigated the influencing factors of reporting behavior from the organizational perspective and identify the paths of communication openness influencing nosocomial infection reporting. This study is meaningful, because it is the first analysis to explore the mediating role of team cohesion in the relationship between communication openness and nosocomial infection reporting through SEM analysis.

Our results confirmed that communication openness had a direct effect on nosocomial infection reporting and communication openness could promote nosocomial infection reporting. Quantitative study showed that pharmacists with good communication openness were 40% more likely to have submitted a medical error reporting, therefore communication atmosphere potentially impacted the likelihood of error reporting, which in turn, could impact patient safety [19]. Medical staff avoided to report publicly adverse events and discussing errors (such as unreasonable aseptic techniques and hand hygiene), possibly due to internal pressure from fear of recrimination or punishment [18, 26]. However, Manojlovich explored the relationship between nurses’ perceptions of elements of communication (one being communication openness) and rates of selected outcomes (pressure ulcers and nosocomial infection), and found that communication openness between physicians and nurses was not related to outcomes [31], which was inconsistent with the results of this study. The possible reason was that due to the differences between Chinese and American cultures, Chinese people were introverted and implicit [32]. Therefore, this study focused on the communication openness of medical staff to promote good outcomes. Therefore, in the IPC management, attention should be paid to improving the free communication and creating the fair and open working atmosphere, that is, encouraging a non-punitive reporting environment [33].

Our study also showed that there was a close relationship between communication openness and team cohesion, which was supported by earlier studies [23, 34] and enriched with quantitative evidences. Previous studies focused on the importance of communication and cooperation in team, which was a broad topic. Our study also found that team cohesion can directly or indirectly influence nosocomial infection reporting, and the effect was remarkable. More specifically, team cohesion directly promoted nosocomial infection reporting. It was consistent with the results of related studies.The results of a meta-analysis revealed stronger correlations between cohesion and performance when performance was defined as behavior [35]. Cross-disciplinary collaboration between medical professionals may contribute to the enhancement of psychologically safe atmosphere and cohesion, so as to be key to preventing nosocomial infection [26]. Previous study has found that leaders play a significant role in promoting team cohesion. Leaders should strive to create an agreeable team climate in which employees are willing to help each other [36]. For instance, Senecal, Loughead, and Bloom provided a plan of team-building interventions through goal setting to enhance team cohesion [37]. Hence, hospital managers should adopt effective intervention to build the cohesion of the medical staff to facilitate the IPC management.

Theses findings clarify the unknown relationship between communication openness, team cohesion and nosocomial infection reporting, since most previous studies focused on the direct role of team cohesion rather than the mediation [26, 35]. Furthermore, previous studies on the mediating effect of team cohesion were mostly reflected in public management and leadership management [14, 38]. For example, cohesion played a mediating role in the relationship between CEO moral leadership and creativity (p < 0.001) [38]. From the perspective of the significant mediating effect of team cohesion, communication openness can improve nosocomial infection reporting to a large extent by team cohesion. However, in the actual work of the hospital, the underreporting of nosocomial infection cases was a common phenomenon [39, 40]. For example, only 8.8% of pharmacists reported suspected cases of COVID-19 symptoms to the health care department during COVID-19 epidemic [41]. The effective way to solve this problem may be to understand how team cohesion is promoted, and pay attention to the mental changes and working atmosphere of medical staff in IPC management. The pharmacy department’s efforts in response to the pandemic have proven successful to this point and have illuminated several lessons, including the necessity of cohesive department communication, teamwork and collaboration [42]. Therefore, team spirit and good collaboration are worthy of attention. It is also necessary to pay attention to communication openness as a pillar to enhance the cohesion of the team, so that improving the quality of the nosocomial infection reporting can achieve the aspiration of IPC.

Although this study helps to establish a knowledge base for nosocomial infection reporting related to communication openness and team cohesion, it does have some limitations. Firstly, the causal relationship between communication openness, team cohesion and nosocomial infection reporting should be carefully explained, because this was a cross-sectional study. Secondly, despite the good reliability and validity, the scale we used was self-designed and required further study. Thirdly, our questionnaire did not contained other potential predictors, which also need further study in the future. Fourthly, due to the use of self-reporting questionnaires, the actual situation about reporting may require the further study. Last, all participants were from Hubei Province, which suggested the generalization of results to other areas may be limited.

## Conclusion

The current findings indicate a positive relationship between communication openness and nosocomial infection reporting, while team cohesion has a significant mediating role between communication openness and nosocomial infection reporting. The mediating effect of team cohesion is 35.03%. Hence, relative measures from the organizational perspective can be taken to promote nosocomial infection reporting thus enhancing IPC.

## Data Availability

The datasets analysed during the current study are not publicly available due to privacy of the infection prevention and management departments in the participating hospitals, but are available from the corresponding author on reasonable request.

## Abbreviations

IPC: infection prevention and control.
SEM: structural equation model.
ADR: adverse drug reaction.
HSOPSC: Hospital Survey on Patient Safety Culture.
LCQ-IP: Leading a Culture of Quality in infection prevention.
